# Benefits of active listening during 3D sound localization

**DOI:** 10.1007/s00221-022-06456-x

**Published:** 2022-09-07

**Authors:** V. Gaveau, A. Coudert, R. Salemme, E. Koun, C. Desoche, E. Truy, A. Farnè, F. Pavani

**Affiliations:** 1 Integrative Multisensory Perception Action & Cognition Team—ImpAct, Lyon Neuroscience Research Center, INSERM U1028, CNRS U5292, 16 Av. Doyen Lépine, BRON cedex, 69500 Lyon, France; 2 University of Lyon 1, Lyon, France; 3 Neuro-immersion, Lyon, France; 4 Center for Mind/Brain Sciences - CIMeC, University of Trento, Rovereto, Italy; 5 ENT Departments, Hôpital Femme-Mère-Enfant and Edouard Herriot University Hospitals, Lyon, France

**Keywords:** Spatial hearing, Virtual reality, Head movements, Motion tracking, Active perception

## Abstract

In everyday life, sound localization entails more than just the extraction and processing of auditory cues. When determining sound position in three dimensions, the brain also considers the available visual information (e.g., visual cues to sound position) and resolves perceptual ambiguities through active listening behavior (e.g., spontaneous head movements while listening). Here, we examined to what extent spontaneous head movements improve sound localization in 3D—azimuth, elevation, and depth—by comparing static vs. active listening postures. To this aim, we developed a novel approach to sound localization based on sounds delivered in the environment, brought into alignment thanks to a VR system. Our system proved effective for the delivery of sounds at predetermined and repeatable positions in 3D space, without imposing a physically constrained posture, and with minimal training. In addition, it allowed measuring participant behavior (hand, head and eye position) in real time. We report that active listening improved 3D sound localization, primarily by ameliorating accuracy and variability of responses in azimuth and elevation. The more participants made spontaneous head movements, the better was their 3D sound localization performance. Thus, we provide proof of concept of a novel approach to the study of spatial hearing, with potentials for clinical and industrial applications.

## Introduction

Spatial hearing is a fundamental ability for humans and other animals. Accurate localization of sounds allows for the construction of maps of the environment beyond the limits of the visual field, guides head- and eye-orienting behavior, plays a crucial role in multisensory integration, supports auditory scene analysis and can improve discrimination of auditory signals from noise. In everyday environments, spatial hearing is three dimensional, multisensory and active. We concurrently estimate azimuth, elevation and distance of sounds, and perceive the visual context in which they occur and often also the event that generated them (Kumpik et al. 2019). Most importantly, in real-life contexts, spatial hearing is an active process: we explore the auditory environment with head and body movements to resolve perceptual ambiguities in sound localization (Andéol & Simpson, 2016).

When spatial hearing abilities are investigated in the laboratory, several of these naturalistic aspects are overlooked. Although this choice is motivated by the aim to control the intervening variables that could contaminate the experimental design, it does not allow full appreciation of some aspects of natural behavior. For instance, although sounds are delivered in 3D and supposedly perceived in 3D space, the response is often limited to one or two dimensions at a time: e.g., participants indicate the angle of the sound source (1D) or its direction (2D) (Andéol and Simpson 2016; Haber et al. 1993; Oldfield and Parker 1984; Wightman and Kistler 1999). In addition, experimental setups often attempt to limit the use of visual cues. Some setups use visible barriers to occlude the sound sources (e.g., Nava et al. 2009; Pavani et al. 2001, 2003), others place participants inside a completely dark room (Goossens and van Opstal 1999; Van Barneveld et al. 2011; Van Grootel et al. 2011), blindfold them (e.g., (Ahrens et al. 2019; Bahu et al. 2016) or require them to keep their eyes closed (e.g., Brungart et al. 1999), thus preventing eye tracking. Yet, eye-orienting responses are relevant for behavior, as the encoding of sound position in retinocentric coordinates has an impact on sound localization (Groh and Sparks 1992; Lewald and Ehrenstein 1996; Maddox et al. 2014; Pavani et al. 2008).

One constraint frequently imposed on participants concerns head position. It is common practice to limit head movements while participants listen to sounds, to ensure reproducible stimulation at the ears across trials and participants. This can be achieved by using a chin rest (Brungart et al. 1999; Litovsky et al. 2009; Pavani et al. 2008, 2008; Távora-Vieira et al. 2015), or by limiting target sound duration to few hundred milliseconds, so that participants do not have time to plan and execute head movements during sound emission (e.g., Ahrens et al. 2019). Even when the response is collected through head movements, these often occur after the sound is finished (e.g., Bahu et al. 2016). These approaches are well motivated when the experimental aim is to achieve full control over the auditory cues reaching the ears. Humans indeed estimate the location of a sound source by combining the auditory cues derived from one ear (monaural cues), with those available at both ears (binaural cues, i.e., interaural level differences ILD and interaural time differences ITD). A rich body of work has examined the specific contributions of auditory cues to sound localization (Middlebrooks 2015). However, limiting head movements during listening brings the drawback that it excludes the contribution of head motion during sound emission in sound localization performance.

In natural listening, head motion is spontaneous and almost ubiquitous. The importance of head movements for sound localization has been remarked since the 1940s (Wallach 1940), with pioneering works in the late 1990s (Perrett and Noble 1997a; Wightman and Kistler 1999). Psychoacoustic studies demonstrate that head motion improves sound localization in humans (Brimijoin et al. 2013; Honda et al. 2013; McAnally and Martin 2014; Perrett and Noble 1997a, b; Pollack and Rose 1967; Vliegen and Van Opstal 2004; Wightman and Kistler 1999), in monkeys (Populin 2006), and cats (Tollin et al. 2005). It is generally assumed that head movements are taken into account during the computation of sound-source coordinate (Goossens and van Opstal 1999). Neck muscle stimulation (Lewald et al. 1999) or cold water in the ear canal (Lewald and Karnath 2000), which alters the perceived position of the head, produces a shift of auditory localization. The continuous integration of head-motion signals (proprioceptive, vestibular and efferent copy signals) contributes to sound localization (Genzel et al. 2016, 2018) and provides a more stable percept of the sound source (Vliegen and Van Opstal 2004).

In the present study, we examined the role of head movements on 3D sound localization by comparing the abilities of adult listeners when their head remains static throughout sound delivery or instead it is free to move (i.e., active listening). Unlike most previous works (e.g., McAnally and Martin 2014), here we focus on spontaneous head movements during sound emission. To this aim, we dissociated possible head movements from the instructed response, which always entailed hand pointing to the sound. In addressing this experimental question, we also developed a novel methodology based on sounds delivered from a free-field sound source (i.e., a loudspeaker) that was continuously tracked and aligned with a virtual reality environment. Specifically, starting from the pioneering work of Brungart and colleagues (1999), we took advantage of current VR technology to guide precisely a loudspeaker to predetermined head-centered coordinates in each trial. Critically, we coupled this head-centered placement of the loudspeaker with criteria for sound emission, based on the participant’s head and eye posture measured in real time. This allowed us to test 3D sound localization abilities without physically restraining the participant’s posture and to assess the contribution of spontaneous head movements to sound localization. We predict better 3D sound localization when participants make spontaneous head movements during listening.

## Methods

### Participants

Twenty normal-hearing participants (range 22–75 years, mean age = 46, SD = 18; 12 females; 18 right-handed) were recruited through advertisements (e-mail or flyer). All reported normal or corrected-to-normal vision and had no history of hearing deficits. Participants were informed that they would participate in a sound localization study that would require wearing a virtual reality HMD and that their task was to localize as accurately as possible a sound delivered in the space around them using a hand-pointing response. If they agreed to participate, they were asked to sign the informed consent documents. The study was approved by the Comité Ethique d’Evaluation de l’Inserm (IRB00003888), and conducted in accordance with the ethical standards of the 1964 Declaration of Helsinki.

#### Apparatus and stimuli

The apparatus was set up in two rooms: a test room and a control room. The control room hosted two desktop computers. The first computer ‘Control PC’, was an HP Z820 Workstation (Windows 7 Professional, Processor Intel(R) Xeon(R) CPU E5-2609 @ 2.40 GHz 2.40 GHz), equipped with a NVIDIA Quadro K5000 graphic card (DirectX 11.0). It controlled the entire sequence of events, stimulations, response collection and data saving through a custom-made script written in Unity (Version 5.5.1f1 32-bit, Unity Technologies, San Francisco, CA). The second computer, hereafter named the ‘Vicon PC’, was an HP Z230 Tower Workstation (Windows 7 Professional, Processor Intel(R) Core (TM) i7-4771 CPU @ 3.50 GHz 3.50 GHz). It controlled the Vicon motion capture system (Vicon Tracker 2.0.1 × 64, Vicon Motion Systems LTD, Oxford, UK) and ran a custom-made script written in Unity that performed stimulus visualization. The test room comprised Vicon cameras for motion capture, three rigid bodies for real-time object tracking, the head-mounted display (HMD) incorporating an eye-tracking system, one monitor for stimulus visualization, one loudspeaker, one keyboard and one remote control. Each of these pieces of equipment is described below, with details concerning the way they were interfaced with the control and Vicon PCs.

*Vicon motion capture*. The Vicon motion capture system comprised seven infrared cameras (Bonita 10: frame rate 250 fps, resolution 1024 × 1024, Vicon^®^, Oxford, UK) mounted on the walls of the testing room. The elevation (195–205 cm) and semicircular arrangement of the cameras allowed full kinematic tracking of a wide 3D space (height: 250 cm; width: 320 cm; depth: 170 cm). The space visible to the cameras was calibrated using the Vicon Active Wand tool (www.vicon.com/products/vicon-devices/calibration), which allows a multi-plane video calibration across the entire acquisition volume. Once calibrated, object-tracking spatial precision was < 1 mm (down to 0, 5 mm in a 4 × 4-m volume). We placed the HMD on the floor in a straight-ahead position to record a straight-ahead reference direction (taking into account HMD rotations). The cameras were connected to a multiport box in the testing room, which in turn was USB connected to the Vicon PC in the control room.

*Rigid bodies*. The Vicon system captured the position of three distinct rigid bodies (each mounted with 4 reflective 9 mm markers), with a sampling frequency of 100 Hz. The first rigid body (rigid body 1; radius 75 mm) was fixed on top of the loudspeaker and tracked its xyz coordinates in the calibrated space; the second rigid body (rigid body 2; radius 75 mm) was fixed on top of the HMD and tracked HMD and the head-center positions; the third rigid body (rigid body 3; radius 75 mm) was used for head-size calibration and for collecting hand-pointing responses.

*Head-mounted display*. The HMD was an Oculus Rift Development Kit 2 system (DK2, Oculus VR^®^, Menlo Park, USA, screen OLED, resolution: 1920 × 1080 (960 × 1080 per eye), maximal refresh of 75 Hz, dimensions L x W x H: 1.3 × 14.7 × 7.1 inches, and a field of view equal to 106°) running with Oculus Runtime (Version 0.6; Facebook Technologies Ireland, Dublin, Ireland). The Oculus Rift DK2 incorporated an eye-tracking system (SensoriMotoric Instruments SMI, Berlin, Germany; (Kuk et al. 2014; Tyler et al. 2010); 60 Hz frequency and 0.5° spatial precision). In our setup, the HMD served two purposes: (1) it conveyed visual instructions to the participant; and (2) allowed continuous monitoring of the participant’s eye movements.

*Loudspeaker.* A loudspeaker (JBL GO portable, 68.3 × 82.7 × 30.8 mm, output power 3.0 W, frequency response 180 Hz–20 kHz, signal-to-noise ratio > 80 dB) was used to deliver all target sound stimuli. Target stimuli were amplitude-modulated broadband bursts lasting 3 s (the sound was modulated at 80%, amplitude varies between 0.2 and 1). The room was a quiet 3 × 6 m place with a reverberation time RT60 of 0.32 s, not treated for being anechoic or soundproof, and the background noise measured at the beginning of the experiment was 33.7 dB SPL. The sound duration of 3 s allows the subject to increase the possibility of moving the head (Thurlow and Mergener 1970) that is beneficial for acoustic dynamic cues (i.e., change in binaural cues ILD and ITD; review by Middlebrooks and Green 1991; Pollack and Rose 1967).

A keyboard, a remote control (Targus^®^, Laser Presentation Remote) and a monitor (DELL 19’’ 5:4, resolution 1280 × 1024), completed the equipment in the testing room. All devices were connected to the control PC, except the stimulus visualization monitor which streamed a copy of the screen of the VICON PC inside the testing room. The function of these four pieces of equipment is described in detail in “Procedure”.

#### Procedure

Before starting the experiment, participants were introduced to the task and to the VR equipment using a visual information sheet. Participants were told that sounds could be delivered anywhere in the 3D space around them at a maximum radius corresponding to their arm-reaching distance, and they would perform the sound localization task under two conditions: ‘static listening’ in which they would have to keep their head still in the initial position throughout sound presentation, and ‘active listening’ in which they could actively search for the sound during its presentation, by freely moving their head. They were also instructed to only pay attention to the sounds, as any other noise in the room could be misleading. In a control experiment (*N* = 6), we ensured that normal-hearing participants were incapable of locating the loudspeaker based on unintentional cues (e.g., experimenter’s displacement), unless the sound was actually emitted. In both conditions, they were free to move their head and body as soon as the sound finished.

The experiment began with eye and head-center calibrations: (i) eye calibration was performed using a five-point calibration grid (smart recorder of SMI Eye tracking software) which permitted control of the 3D cyclopean eye position and direction; (ii) head-center calibration was performed by collecting the 3D position of the two ears (using rigid body 3), averaging these positions to obtain the 3D head-center position. The head-center position served as the origin for the polar coordinate system that included loudspeaker, hand, head and cyclopean gaze positions. In this way, even though participants sat without a chin rest, we could carefully control the position of each sound source with respect to their head position. Twelve predetermined positions were used throughout the experiment, resulting from the combination of four different azimuths (− 30°, 30°, − 150° or 150°), three different depths (35 cm, 55 cm or 75 cm) and a single elevation (0°, i.e., ear level). Despite that the elevation remained constant, participants were left unaware of the 3D sound position and had no visual cue that constrained the possible sound origin. Moreover, their response could vary in all dimensions, thus rendering their response elevation also relevant to this experiment. For these reasons, we studied the participants’ responses in 3D.

**Fig. 1:**
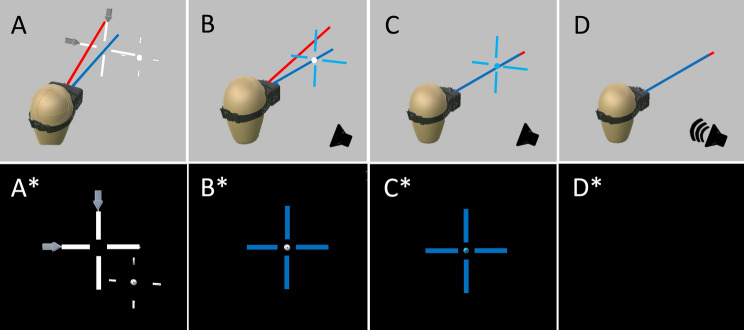
Pre-stimulation alignment of head and eyes. **A** At the beginning of each trial, the participant was free to move their head (symbolized head direction: blue line) and eyes (symbolized cyclopean eye direction: red line). A* In the HMD display: a bold white cross indicates the actual position of the head, and a thin white cross with a central ball indicates the desired position of the head and eyes, respectively. Two gray arrows flank the bold white cross, showing the participant in which direction to move their head to achieve the desired initial position. B* When the desired head position was achieved, the bold cross turned blue. C* When the desired eye-gaze position was reached, the central ball turned blue. **D** When all criteria were met (head and eye position straight ahead and speaker within a sphere around the predetermined position), all visual stimulations were removed, D* the scene became dark and the sound was delivered

In each trial, two sets of instructions, generated in real time by the computer, informed the experimenter where to position the loudspeaker in the 3D space surrounding the participant. The stimulus visualization monitor displayed in real time and in 2D (azimuth and depth) the actual position of the loudspeaker and its desired position for the upcoming trial. The precise elevation positioning of the speaker was communicated to the experimenter via an echo radar sound delivered by an in-ear headphone (non-audible by the participant). This allowed the experimenter to rapidly place the loudspeaker in a predetermined position with a margin of 3D error of 2.5 cm. Sound could only be delivered when three criteria were concurrently met: (1) the loudspeaker was in the correct 3D position; (2) the participant’s head was facing straight ahead; (3) the participant’s eyes were directed straight ahead. Participants actively complied with criteria 2 and 3 by aiming their head and eyes to align two crosshairs displayed in the HMD (cf Fig. 1). At the end of sound emission, the experimenter removed rapidly the loudspeaker out of the emission area so that participants did not collide with it when pointing with their hand-held rigid body. After trial completion, no feedback on performance was ever provided.

The experimental session was organized into four successive blocks. Listening conditions (static or active) changed between blocks of trials (half of the participants followed an active–static–static–active sequence, whereas the other half followed a static–active–active–static sequence). Each block consisted of 48 randomized trials, resulting in a total of 192 trials (8 trials × position × listening condition).

Each participant completed the whole experiment in approximately 40 min. Each trial lasted 10–15 s, with the speaker-positioning phase lasting 3–5 s, depending on the predetermined position. Multiple aspects contributed to trial duration: events before sound delivery (the participant actively moved head and eyes to the desired initial posture), the experimenter manually bringing the loudspeaker to the predetermined position, the sound delivery itself (3 s), and the participant’s full body motor response (mean reaction time for the hand responses was 3.65 s ± 0.48). No time restrictions on the response were imposed.

#### Data processing

The position of all tracked elements recorded in Vicon reference frame (loudspeaker, head center and direction, hand) was re-computed in head-center reference frame. Kinematic analyses of head and hand were conducted and inspected for each trial using an in-house software running on MATLAB R2013a, which allows to filter, identify and scrutinize head and hand movements in the kinematic trace (for previous description of this procedure, see (Gaveau et al. 2008). Specifically, head and hand position signals were first filtered (50 Hz cutoff frequency, finite impulse response filter FIR) and velocities were computed from the filtered position signal using a two-point central difference derivative algorithm (Bahill and McDonald 1983). To obtain the spatio-temporal profile of head behavior and to extract relevant parameters for subsequent analyses, the beginning and the end of all movements were automatically detected using a velocity threshold procedure (80 mm/s). The number of head movements during sound emission corresponds to the number of times the speed threshold was exceeded. This procedure was also applied to hand movements. Finally, we extracted relevant kinematic parameters for subsequent analyses.

To evaluate overall sound localization performance in 3D (i.e., across azimuth, elevation and depth, and irrespective of sound position), we computed a cumulative index, called the 3D error, for each listening condition. To do this, we adapted the error calculation proposed by Rakerd and Hartmann (Rakerd and Hartmann 1986), which combines into a single measure the absolute constant error (referred to as $\underline{C}$) and the random error as follows: $\sqrt{\underset{\_}{{C}^{2}}+\underset{\_}{{s}^{2}}}$. To obtain overall error in 3D (3D error), we calculated for each trial $i$ the norm of the vector $\underset{\_}{{C}_{i}}$. This is the distance in 3D space between the participant’s response (i.e., the coordinates of the rigid body held in the participant’s hand, ${x}_{h}$, ${y}_{h}$, ${z}_{h}$) and the speaker location at the moment sound was delivered (i.e., the coordinates of the rigid body mounted on the speaker, ${x}_{s}$, ${y}_{s}$, ${z}_{s}$). All values of $\underset{\_}{{C}_{i}}$ extracted for each participant were then averaged irrespective of sound position. The random error for each participant was computed as the standard deviation of the responses at each sound position, averaged across all sounds.

All statistical analyses and data visualizations were performed using R in the R-studio environment. We used ‘afex’ package for ANOVA designs and the ‘lsmeans’ package for estimating marginal means and run comparisons. We also used ‘dplyr’ and ‘Rmisc’ for preprocessing and ‘ggplot2’, ‘ggpubr’ and ‘cowplot’ for data visualization. Unless otherwise indicated, means ± standard errors are reported in the text. We planned ANOVAs or *t *tests, and the Greenhouse–Geisser sphericity correction was applied to analyses of variance, when appropriate. The Holm–Bonferroni method was applied to adjust of the *p* values for multiple comparisons (Midway et al. 2020). Data for all statistics, complete analysis pipeline and scripts are available in the supplementary materials and at the following link: osf.io/8fapq.

## Results

### Positioning the loudspeaker at predetermined locations

In the absence of physical constraints on participant posture, the head returns to slightly different initial positions at the beginning of each trial. This poses a potential problem of reproducibility of sound source positioning across trials and participants. Participants were instructed at the beginning of each trial to align their head and eyes with respect to straight-ahead reference, using visual cues available in the HMD. Online head kinematics tracking allowed sound to be delivery only when the required eye and head posture criteria were matched. In addition, and most importantly, on each experimental trial, we guided the loudspeaker to a predetermined location in the environment defined in head-centered coordinates.

**Fig. 2:**
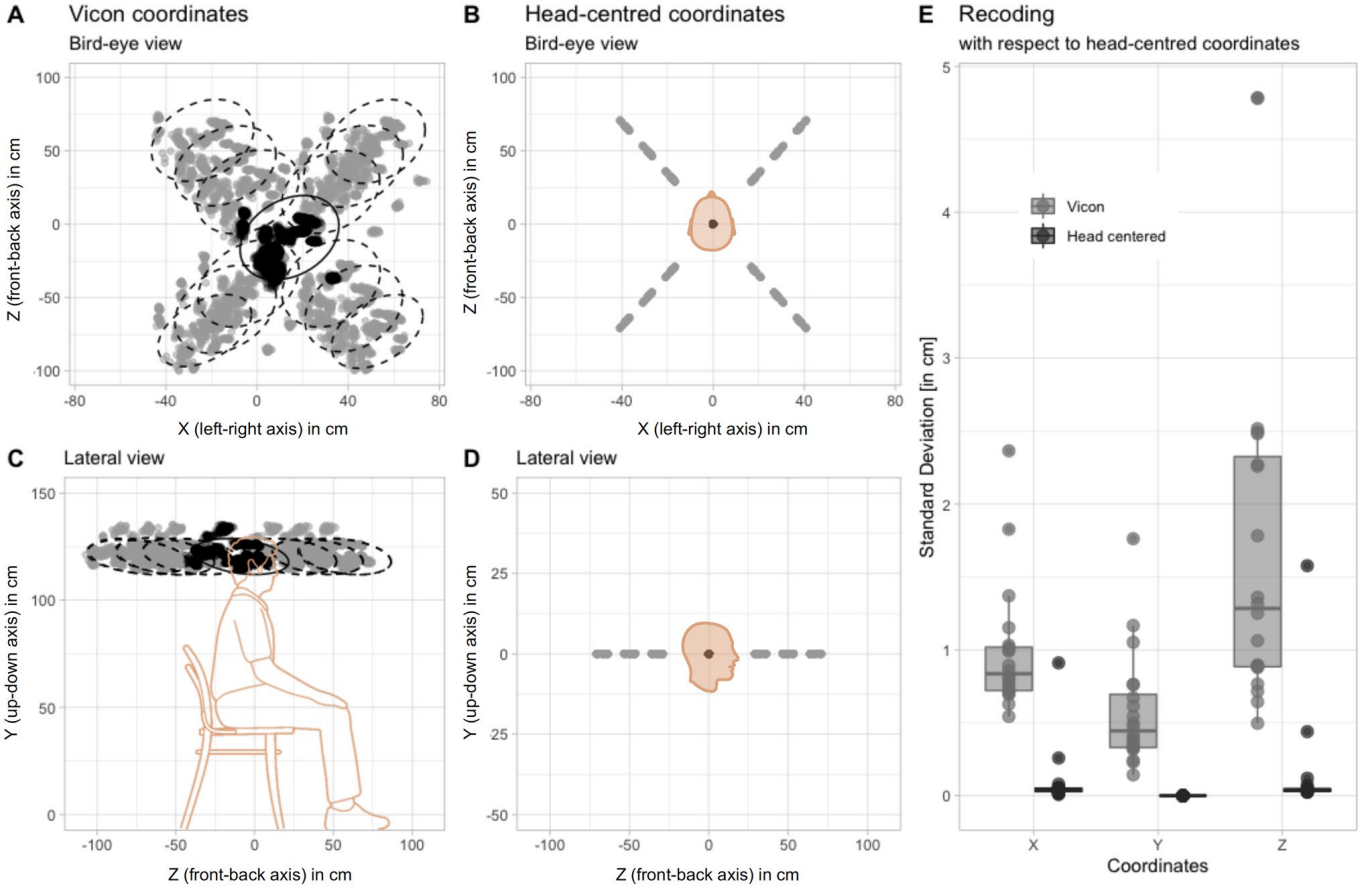
Normalization to head-centered coordinates. Bird’s-eye and lateral views of initial head position (in black) and 12 predetermined locations (in gray) for all participants (192 trials each), in VICON reference frame (**A**–**C**) and head-centered coordinates (**B**–**D**). Variability of predetermined locations averaged across 12 positions for each participant in the two reference frames as a function of coordinates *x*, *y*, *z* (**E**)

**Fig. 3:**
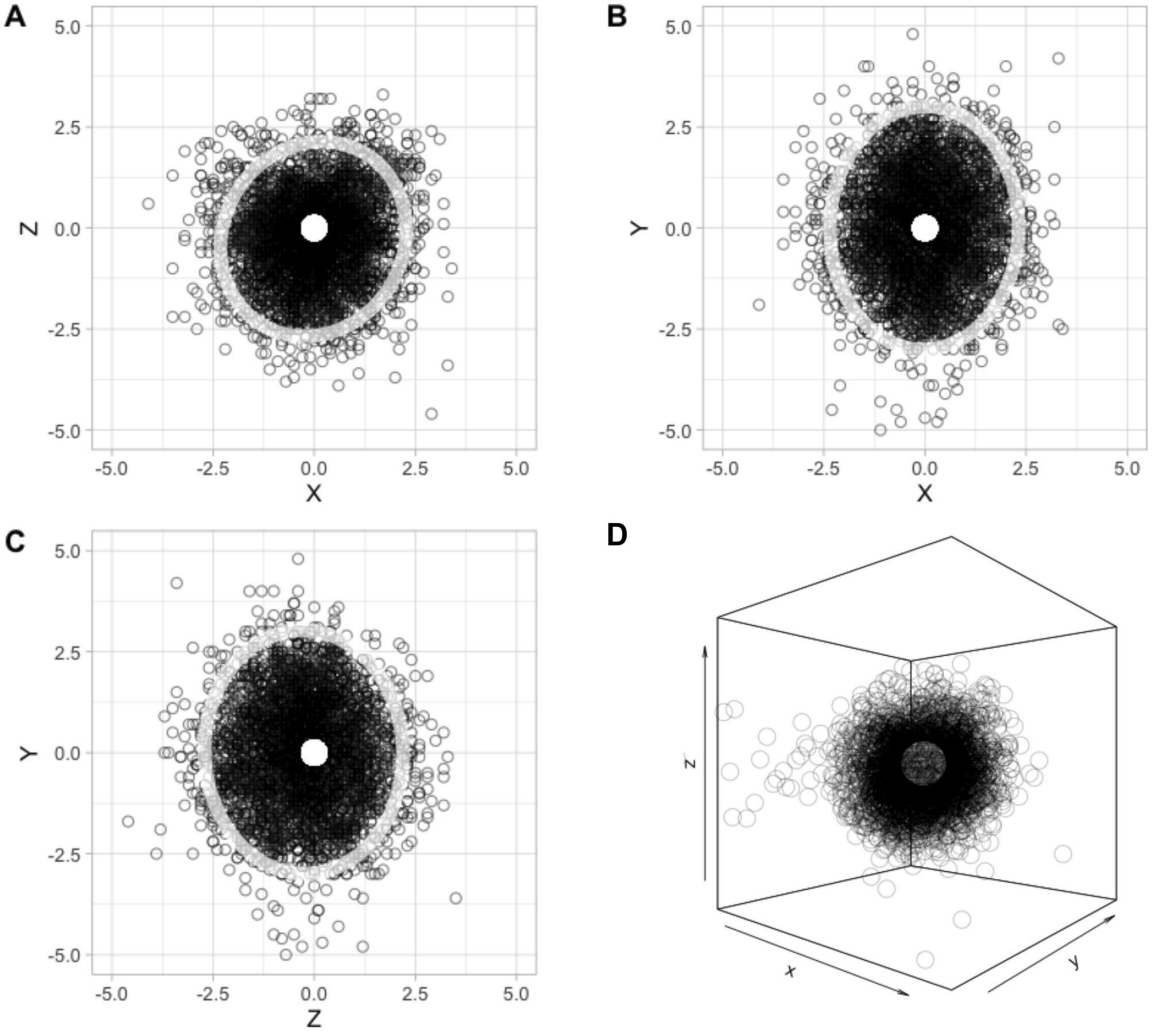
Actual speaker location with respect to predetermined location, in centimeters. Stimulations delivered to all participants, when the 12 predetermined locations were re-aligned to a single coordinate, centered on the origin of the axes. **A** top view; **B** front view; **C** lateral view; **D** 3D rendering. Ellipses in the 2D panels represent 95% confidence intervals of the distributions

We started by assessing if the predetermined loudspeaker locations remained constant across trials and participants. We tested 12 sound locations all around the participant’s head. Figure 2A and C shows initial head position and predetermined locations for all participants across all trials, in the reference frame of the motion-tracking system (i.e., Vicon coordinates). A substantial variability is observed, which reduces dramatically, however, when all positions are referenced to the center of the head, i.e., they are converted to a head-centered reference frame (Fig. 2B and D). Figure 2E summarizes the effect of head-centered referencing by comparing mean changes in standard deviation across participants in motion-tracking vs. head-centered coordinates (*x*: 1.08 ± 0.24 cm vs. 0.10 ± 0.02 cm; *y*: 0.63 ± 0.14 cm vs. 0.10 cm ± 0.0; *z*: 1.89 ± 0.42 cm vs. 0.15 ± 0.03 cm);

Next, we tested if the variability of loudspeaker actual location around the predetermined position was within the established tolerance (i.e., a sphere with a 2.5 cm radius around the predetermined position). Indeed, this was the case. Figure 3 shows all 192 stimulations for all participants when the 12 predetermined locations were re-aligned to a single coordinate, centered on the origin of the axes (mean differences with SE between predetermined and actual location: *x* = 0.87 ± 0.01 cm, *y* = 1.15 ± 0.02 cm, *z* = 0.93 ± 0.01 cm; error in 3d = 1.98 ± 0.01 cm).

During sound emission, all visual references for the straight-ahead head posture in the HMD were removed. Depending on the experimental conditions, participants were either instructed to keep their head motionless (static listening condition) or they were free to move their head and explore space (active listening condition). Irrespective of the listening conditions, participants were free to move their body and indicate the 3D sound location with their hand as soon as the sound ended. Participant compliance with the instructions was examined off-line and trials in which instructions were not followed (e.g., anticipatory hand or head movements during sound delivery in static condition) were excluded from further analyses (static listening: 6.1%, SD = 8; active listening: 6.6%, SD = 7). The main reason for trial rejection was anticipatory hand responses.

Thus, our apparatus based on head-centered positioning of a loudspeaker in the environment allowed high precision 3D control of sound position with a minimal constraint on participant posture.

### Sound localization during static and dynamic listening

**Fig. 4:**
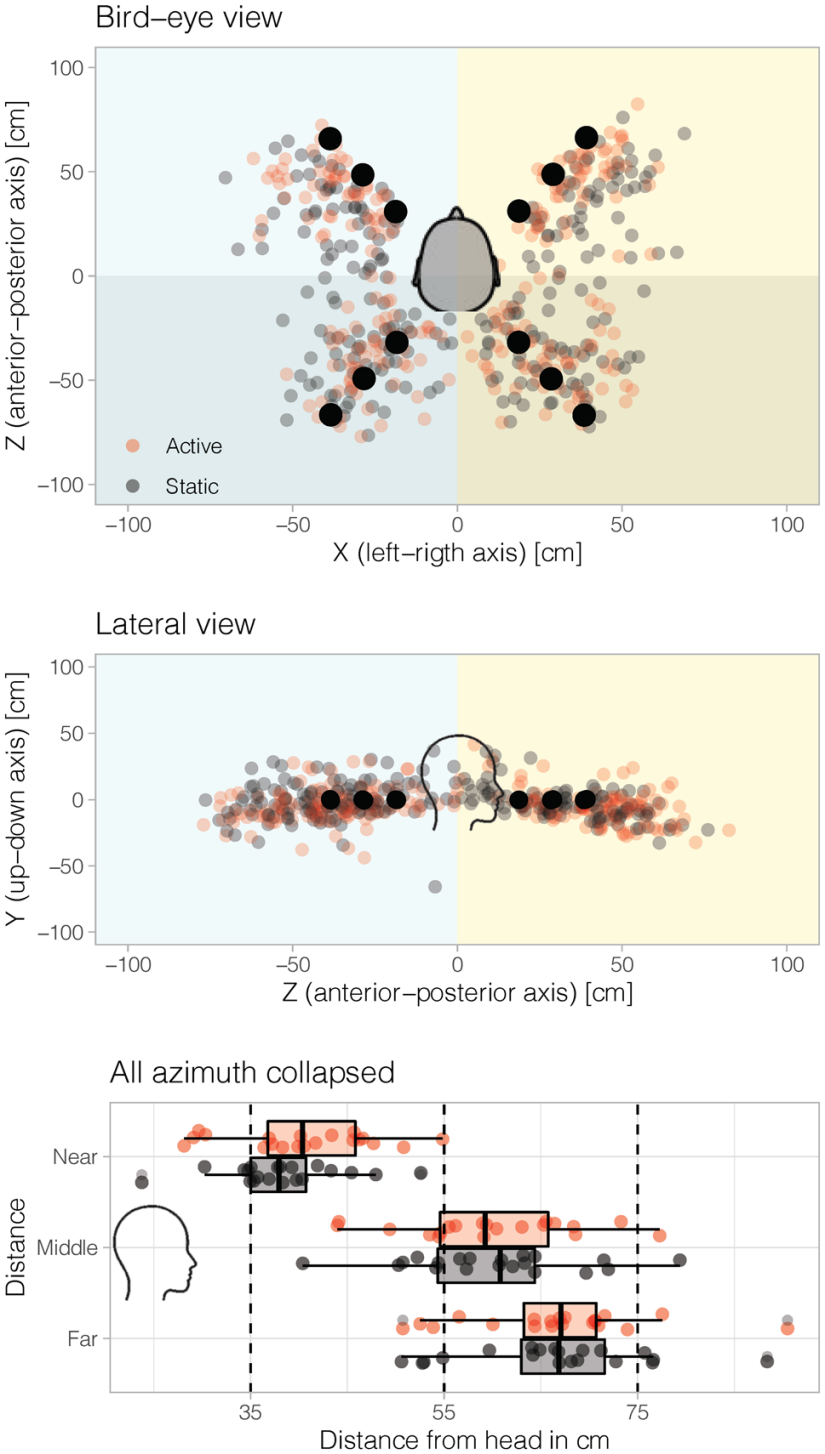
Behavioral pointing and effects of static and active listening on sound localization. **A** Bird’s-eye view of all target positions (black dots) and hand-pointing responses (smaller gray and red circles) for each participant, averaged across trials in a quadrant (i.e., front-left, front-right, back-left, back-right) irrespective of sound distance. Color code is a function of listening condition (black: static listening; red: active listening). **B** Lateral view of all target positions and responses. Responses for each participant are averaged across (left or right) and distance (near, middle or far). **C** Lateral view of responses in depth (black box plot: static listening; red box plot: active listening). All participants were included

In this study, we wanted to examine whether active listening (i.e., free head movements during sound presentation) changed spatial hearing performance (Fig. 4 depicts hand-pointing responses in each separate dimension).

**Fig. 5:**
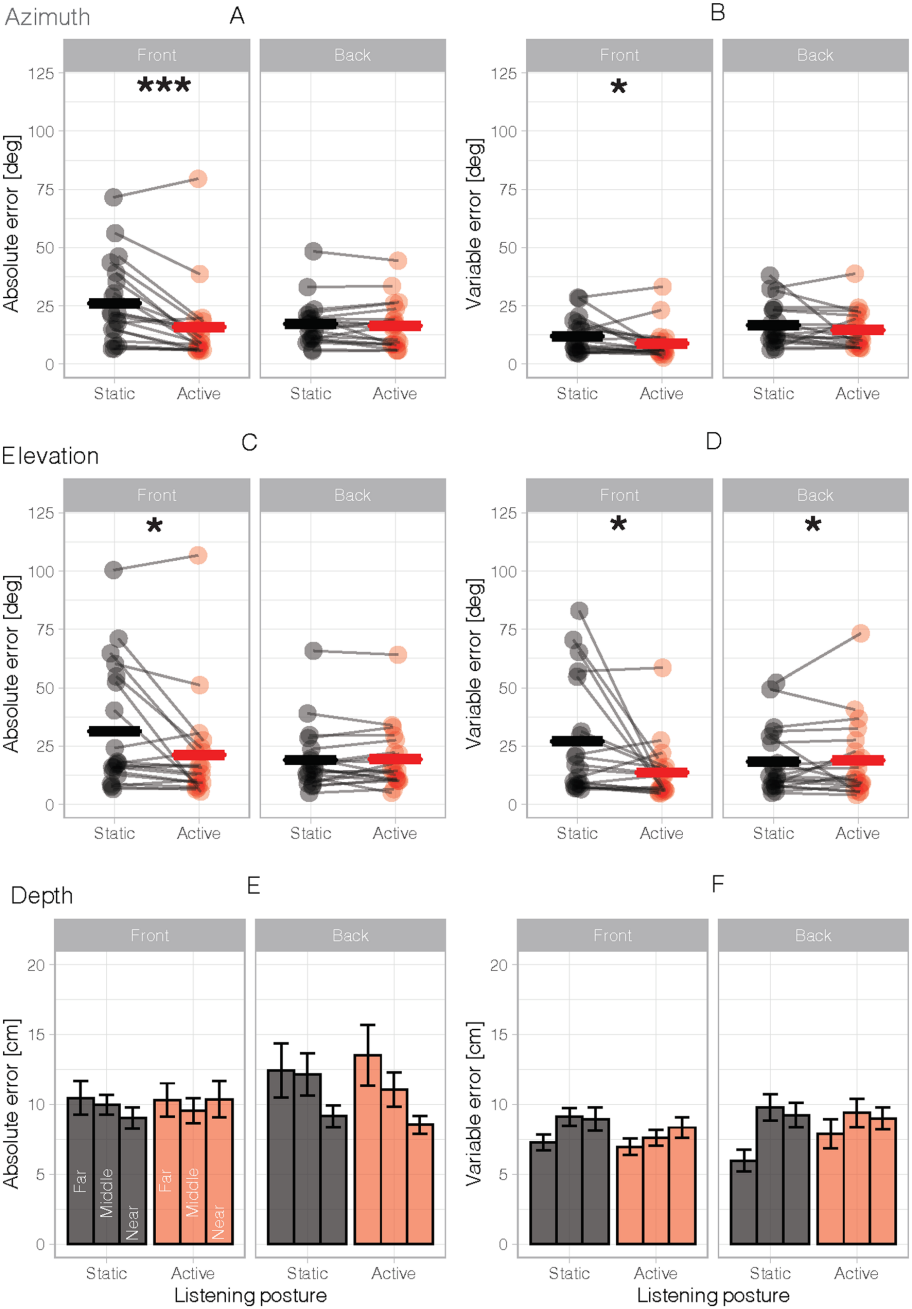
Effects of static and active listening on sound localization performances. In azimuth dimension, for the hand absolute error (**A**) and the hand variable error (**B**) for each participant as a function of listening condition and antero-posterior position of target sounds. In elevation dimension, for the hand absolute error (**C**) and the hand variable error (**D**) for each participant. In depth dimension, for the hand absolute error (**E**) and the hand variable error (**F**) for each participant and for the three distance (near, middle and far sound position). Bold horizontal lines indicate the mean for all participants. Asterisks indicate significant differences (* *p* < 0.05; **p < 0.01; ***p < 0.001)

The effect of the listening condition was evaluated for the three dimensions (azimuth, elevation and depth). Figure 5 presents changes in absolute and variable localization errors as a function of listening condition; the absolute error reflects the accuracy of the performance and the variable error the precision.

To study azimuth errors along the antero-posterior axis as a function of listening condition, we entered absolute and variable errors in separate ANOVAs with antero-posterior sector (front and back) and listening condition (static and active) as within-participant factors. The analysis on azimuth absolute errors revealed an effect of listening condition (F(1,19) = 17,58, p <  = 0.001, $n_{g}^{2}$  = 0.04). The error reduction occurred in front space for active (15.9 ± 3.8°) compared to static listening (26.1 ± 4.0°, *p* = 0.0005), whereas no such change occurred in back space (static: 17.2 ± 2.2°; active: 16.5 ± 2.3°; *p* = 0.43; Fig. 5A). The expected two-way interaction between the antero-posterior sector (front and back) and listening condition (static and active) was significant (*F*(1,19) = 12.93, *p* = 0.002, $n_{g}^{2}$ = 0.03). Azimuth variable error also benefited from active listening and was thus reduced in active (11.7 ± 1.4) compared to static listening (14.3 ± 1.3) (*F*(1,19) = 4.55, *p* = 0.046, $n_{g}^{2}$ = 0.03), and in front space (10.4 ± 1.4) compared to back space (15.6 ± 1.7) (*F*(1, 19) = 6.06, *p* = 0.024, $n_{g}^{2}$ = 0.10), without interaction between these two factors (listening condition and antero-posterior sector, *F*(1, 19) = 0.20, *p* = 0.660, $n_{g}^{2}$ < 0.01) (Fig. 5B).

A convergent result emerged for elevation. When absolute and variable elevation errors were entered into an ANOVA similar to the one described above. Elevation absolute errors in front space were reduced for active (21.2 ± 5.1°) compared to static listening (31.3 ± 6.0°, *p* = 0.02), whereas no such change occurred in back space (static: 19.0 ± 3.1°; active: 19.3 ± 3.0°; *p* = 0.7; Fig. 5C). The two-way interaction between antero-posterior sector and listening condition reached significance for absolute errors (*F*(1,19) = 6.54, p = 0.019, $n_{g}^{2}$ = 0.02). Again, elevation variable errors were reduced in active (16.2 ± 2.6) compared to static listening (22.6 ± 3.2), irrespective of whether stimuli were in front or back space (main effect of listening condition for the antero-posterior sector, *F*(1,19) = 4.59, *p* = 0.045, $n_{g}^{2}$ = 0.03, Fig. 5D), with an interaction between listening condition and antero-posterior sector, (*F*(1, 19) = 6.06, *p* = 0.024, $n_{g}^{2}$ = 0.04).

By contrast, active listening did not affect depth estimation. When absolute errors in depth were entered into an ANOVA with distance (near, middle, far), antero-posterior sector (front and back) and listening condition (static and active) as within-participant factors, no significant main effect or interaction involving listening conditions emerged (all Fs < 2.97, Fig. 5E). Likewise, no main effect or interaction involving listening condition emerged for variable errors in depth (Fig. 5F), and we noticed only a main effect of the antero-posterior sector (*F*(1,19) = 6.69, *p* = 0.018, $n_{g}^{2}$ = 0.02) without interaction with the listening condition. Participants were able to perceive three distinct depth positions (hand distance from the head for near, middle and far sound position, 39.4 cm, 60.2 cm and 66.4 cm respectively; *F*(1.19, 22.63) = 176.13, *p* < 0.001, $n_{g}^{2}$ = 0.62).

Taken together, these results show that active listening (free and spontaneous head movements) improved sound localization (accuracy and precision) in azimuth and elevation.

### Head movements during active listening

**Fig. 6:**
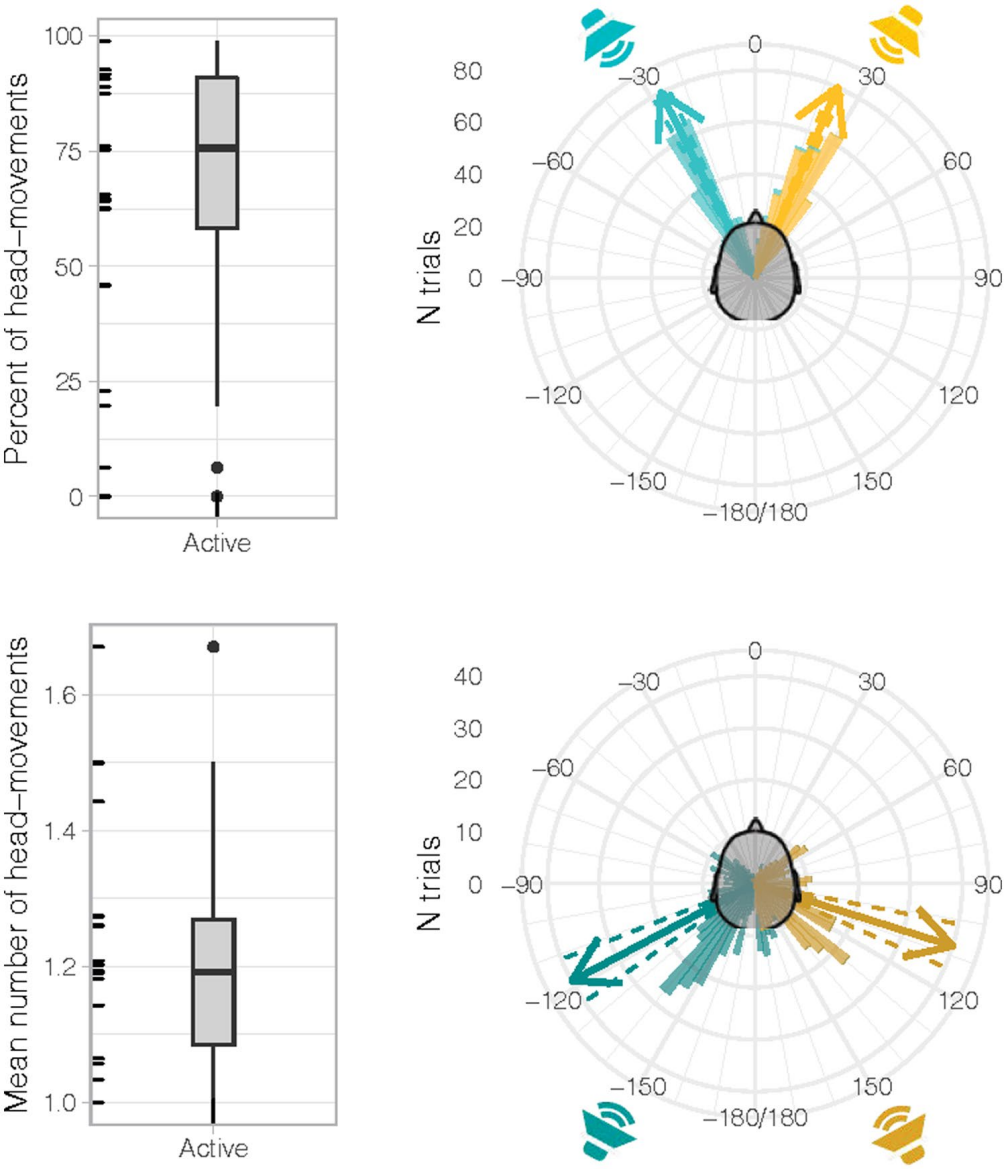
Head movements during sound emission in the active listening condition. **A** Box plot of percentage head movements. Note that two participants were identified as outliers (i.e., they fell outside the 1.5 × interquartile range), made almost no head movements during the active listening condition and were thus excluded from subsequent analyses. **B** Box plot of mean number of head movements once outliers were removed. **C**, **D** Polar histogram showing the distribution of head-movement responses for targets in front (**C**) and back (**D**) space. Arrows indicate mean head-movement direction, dashed lines indicate ± 1 SE

Continuous kinematic tracking of the HMD allowed detailed investigation of head movements during sound emission in the active listening condition (recall that participants were only told that head movements were possible, they were not explicitly instructed to move their head upon sound presentation or to orient to the sound with their head. Likewise, during the response phase it was made clear that only hand-pointing was relevant for measuring performance). Even though the active listening condition allowed free head movements during sounds, not all participants moved their head. As visible in Fig. 6A, the distribution of percent head movements revealed two outliers (i.e., points beyond 1.5 of the interquartile range, IQR): one participant who never moved his head and another who moved only in 6 out of 96 trials (6.3%). These two outliers were removed from all subsequent analyses on head movements.

The mean number of head movements during sound was 1.22 ± 0.04, with an average onset at 1077 ± 73 ms (head movements beyond 3000 ms, i.e., after sound emission, were removed from this analysis; Fig. 6B). Head movements occurred on 73.6% of trials on average (SD = 24.0%), both for targets in front and back space (74.7% and 72.5% of trials, respectively). For targets in front space, they were mostly directed to the target. On average, for sounds at + 30°, the first head movement was directed to 23.6 ± 1.8°, whereas for sounds at − 30° it was directed to − 27.0 ± 2.5° (Fig. 6C). For targets in back space head movements were distributed within the entire stimulated hemispace (Fig. 6D). They were either directed to the front quadrant on the same side as the target (e.g., left front quadrant for targets at − 150°) or aimed directly at the back target (in this case involving a trunk movement). On average, for sounds at + 150° the first head movement was directed to 107.5 ± 6.3°, whereas for sounds located at − 150° it was directed to − 118.0 ± 6.6.

### 3D error

**Fig. 7.**
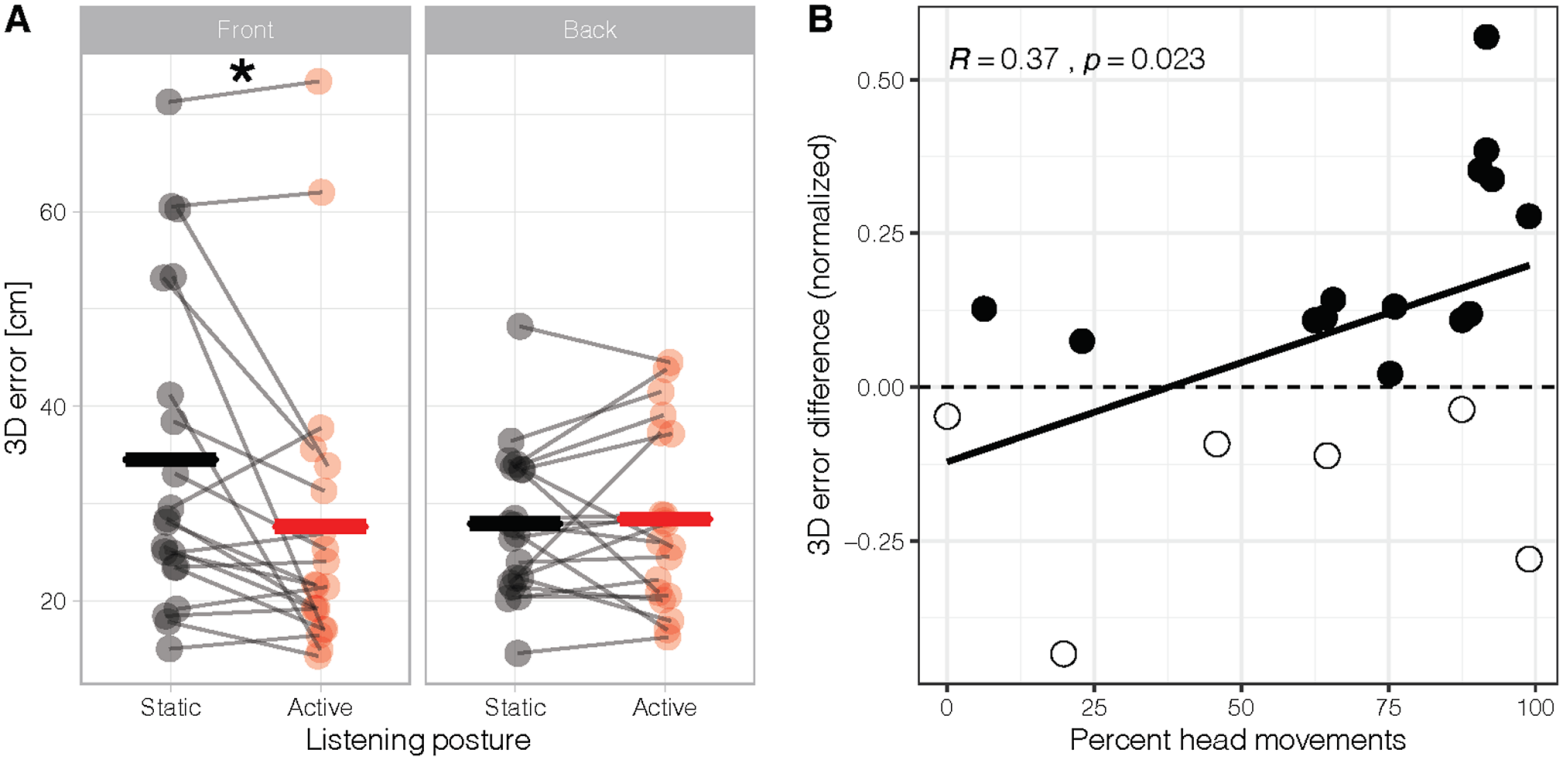
3D error. Scatterplot of the difference in 3D errors between active and static listening conditions (normalized difference based on static listening performance), as a function of percent head movements. Filled circles indicate participants who improved in active compared to static listening; empty circles indicate participants whose sound localization performance decreased in active compared to static listening

As a final step, we quantified overall sound localization performance in 3D using the 3D error (see “Data processing” for details). Figure 7 shows change in 3D error in the two listening conditions. Considering all participants and trials, we ran an ANOVA with antero-posterior sector (front and back) and listening condition (static and active) as within-participant factors in 3D hearing performance. The improvement in sound localization in active (28.0 cm ± 2.3) compared to static listening (31.2 cm ± 2.1) emerged as marginally significant (*F*(1,19) = 3.97, *p* = 0.061, $n_{g}^{2}$ = 0.02), but was significant in front space when taking into account listening posture (active = 27.6 ± 3.4 cm, static = 34.5 ± 3.7 cm; *F*(1,19) = 7.67, *p* = 0.012, $n_{g}^{2}$  = 0.02). When the difference between active and static posture was studied as a function of the mean number of head movements, a positive correlation emerged (*r* = 0.37, *p* = 0.023, Kendall’s rank correlation tau). The higher the proportion of trials with head movements during sound emission, the greater was the performance improvement in active compared to static listening. A convergent correlation emerged also between the mean number of head movements and 3D error (*r* = 0.34, *p* = 0.038).

## Discussion

In the present study, we examined to what extent spontaneous head movements improve sound localization in 3D—azimuth, elevation, and depth—by comparing static vs. active listening postures. To this aim, we developed a novel approach to sound localization based on sounds delivered in the environment brought into alignment with a VR system. Our system proved effective for the delivery of sounds at predetermined and repeatable positions in 3D space, without imposing a physically constrained posture, which required minimal training. In addition, it allowed measuring participant behavior (hand, head and eye position) in real time.

### Active listening improves 3D sound localization

In the static listening posture, normal-hearing participants reliably discriminated sound sources in azimuth, elevation and distance. Absolute errors were 21.6°, 25.2° and 10.5 cm on average, respectively. Along the horizontal dimension, performance in azimuth was worse for front (26.1°) compared to back targets (17.2°), with a bias to point to more eccentric positions for frontal sources. Likewise, for elevation, inaccuracies were more evident for frontal sources compared to rear ones. It is worth mentioning that the overall angular error measured in our study is numerically greater than those obtained by other studies. For comparable elevation position, Brungart and colleagues (1999) obtained a mean angular error of 16.3°, and Wightman and Kistler (1999) a mean error of 21.1°. Of particular interest are errors in depth, which are typically much less investigated compared to those in azimuth and elevation. In the present work, participants succeeded in distinguishing the three sound sources in depth, but underestimated far targets compared to near ones. This is a well-established pattern when studying depth perception in spatial hearing (Brungart et al. 1999; Kearney et al. 2010; Kolarik et al. 2016; Middlebrooks and Green, 1991; Parseihian et al. 2014; Zahorik 2002; Zahorik et al. 2005; Zahorik and Wightman 2001). Sound distance cues, such as interaural level difference (ILD) and direct-to-reverberant energy ratio in reverberant environments are important for distance localization in the near-head acoustic field (Kopčo and Shinn-Cunningham 2011; Kolarik 2016; Brungart et al. 1999). In the near field specifically, sound distance perception also relies on low-frequency ILD (Brungart et al. 1999; Middlebrooks and Green 1991) and the closer the sound is to the listener, the more does the ILD contain low frequencies, and therefore the more does the distance accuracy increases (Brungart et al. 1999; Kolarik et al. 2016). Some others factors may increase this degree of precision, such as the presence of an echoic room (see review from Kolarik et al. 2016) and the lateral positioning of sound sources (Kopčo and Shinn-Cunningham 2011). Thus, our participants may have benefited from several effective cues to distinguish the different distances.

While performance of normal-hearing adults may appear relatively inaccurate in 3D (overall), it is important to consider three aspects of our paradigm that may have contributed to this outcome. First, at odds with most previous studies, here participants' 3D errors combine uncertainty across all three dimensions (i.e., azimuth, elevation and distance, all unknown to participants). Second, participants had to resolve sound position in a totally dark 3D space. Concurrent estimation of the three space dimensions may have been particularly difficult in the absence of visual references, especially in front space where visual cues typically contribute to perceived sound position (Alais and Burr 2004). Third, wearing an HMD may have altered sound localization cues. The HMD, which acted as a physical obstacle to sound diffusing from front near (Ahrens et al. 2019; Genovese et al. 2018; Gupta et al. 2018). For instance, Ahrens and colleagues (2019) documented larger azimuthal errors for lateral sound sources delivered in front space, when participants used the HMD compared to when they localized sounds without it).

In the active listening posture, no specific head-movement strategy was imposed to participants who were free to move their head or not during sound emission. As a matter of fact, most participants engaged in active listening (only two participants moved their head only in a few trials or not at all), which improved 3D sound localization primarily by ameliorating accuracy and variability of responses in azimuth and elevation. The more participants made spontaneous head movements, the better was their 3D sound localization performance. The benefit of active listening emerged selectively in the front space. As this is the portion of space in which participants were less accurate and less precise in the static condition, they may have leveraged a greater margin for their improvement.

Wallach (1940) was the first to report benefits of head movements for spatial hearing. Other works showed that head movements help normal-hearing listeners to distinguish between sounds coming from front and rear positions (Dunai et al. 2011; Mueller et al. 2014; Perrett and Noble 1997b; Wightman and Kistler 1999). A more recent study (Kim et al. 2013) compared azimuthal sound localization under conditions of active head movements, passive head movements, and body movements with the head fixed. The results of Kim and colleagues (2013) suggest that vestibular information associated with head movements may be both necessary and sufficient to improve sound localization. In contrast, proprioceptive information alone (available in the body movement with the head-fixed condition) does not improve localization. The impact of head movements on sound localization abilities has been recently documented also in adults and children with hearing loss who use cochlear implants. Pastore and colleagues (Pastore et al. 2018) showed that front–back confusions diminish in bilateral cochlear implant users asked to rotate their head within a range of approximately  ± 30°, compared to a static head posture. Similarly, Coudert and colleagues (2022) found that sound localization in 3D improves when children with bilateral cochlear implants are allowed to spontaneously move their head (as here), compared to a static head posture.

For depth perception, active listening did not change performance accuracy or variability, in front or back space. Wearing an HMD did not alter the low-frequency ILD component of sound (Ahrens et al. 2019), the position of sound sources (close to the head and lateral) and reverberation cues likely yielded enough localization cues to solve the distance discrimination task without help of head motion.

The functional mechanisms that underlie sound localization improvements by head movements remains to be ascertained. On the one hand, the active listening benefit could result from richer auditory cues at the ears (i.e., at the auditory processing periphery). Head rotations, either horizontal (left–right) or vertical (head tilted up or down) inevitably produce dynamic acoustics cues (change in binaural cues) that, in turn, could facilitate sound localization (Lambert 1974; Perrett and Noble 1997b). In addition, head movements cause the interplay of sensory and motor signals (proprioception/efference copy and vestibular), which might be better integrated with dynamic binaural cues to solve the sound localization task. On the other hand, intentional head movements make sound localization a predictive process. Participants could benefit from ‘hypothesis verification through action’: predictions about sound location are constantly updated based on the incoming error signals that result from head movements, resulting in an interactive cycle that generates a more veridical model of the auditory environment (see Yon et al. 2019) for related examples from the visual modality). Notably, this central mechanism could remain valid also when peripheral auditory information is less accurate (as in the case of people with hearing loss, or using hearing aids or cochlear implants).

### Pursuing active sound localization in 3D: a methodological challenge

Pursuing active approaches to sound localization in 3D space may be a shared objective when aiming to measure and capture the complexity of this fundamental behavior in real life. Yet, it remains a methodological challenge to achieve this aim in research and clinical settings. Below, we briefly discuss alternative approaches to this problem and summarize the advantages and limitations of the novel methodology we have introduced.

#### Multiple loudspeakers in the physical environment

One approach to the study of sound localization in 3D space is with multiple loudspeakers placed at fixed locations in the physical environment (e.g., Ahrens et al. 2019; Bahu et al. 2016). With these experimental setups, it is practically mandatory that the participant keeps a fixed posture at the beginning of each trial, because this is the only way to ensure replicable positions of target sounds with respect to the ears (Bahu et al. 2016; Brungart et al. 1999; Oldfield and Parker 1984; Seeber et al. 2004; Wightman and Kistler 1992). However, this often implies that participants also keep a fixed posture throughout the trial. In addition, because sound sources are physically present, these approaches face the problem of controlling the contribution of visual cues to sound localization. Participants are sometimes blindfolded from the moment they enter the experimental room (Ahrens et al. 2019; Bahu et al. 2016), or they are instructed to close their eyes at specific moments during the task (Brungart et al. 1999), or face speakers hidden behind a fabric panel of some sort (Rabini et al. 2019). Note that the first two solutions pose the problem that they prevent tracking of eye position. In natural conditions, eye-orienting responses permit encoding of sound position in retinocentric coordinates (Bulkin and Groh 2006; Pavani et al. 2008), and it has been documented that static and dynamic eye position influence sound localization (see Groh and Sparks 1992; Lewald and Ehrenstein 1996; Pavani et al. 2008). Our approach allows to control for initial eye position, while continuously monitoring the listener’s head position rather than asking participants to close their eyes and stay still in a predetermined position, and allows control of visual cues.

Brungart and colleagues (1999) were the first to have the intuition of tracking the kinematic of a single loudspeaker, displaced trial-by-trial at different locations around the listener head, to study spatial hearing in 3D. Although the speaker position in each trial was somewhat approximate (the experimenter received verbal instructions about the predetermined speaker location through headphones), its actual location was recorded at the end of each trial using a position-sensing system mounted on the chin rest. Using this sound method, Brungart and colleagues (1999) succeeded in placing sound sources in 3D space. While innovative, this experimental setup was complex and time consuming. Participants had to familiarize with the procedure before data collection. Moreover, the method had intrinsic limitations. First, sound source positions were variable among participants because the speaker’s coordinates were interpreted by the experimenter in each trial using a number-to-coordinate mapping. Second, participants had to close their eyes during sound positioning, thus limiting most oculomotor information that could have enhanced sound localization abilities (Maddox et al. 2014). Third—and most important—to ensure reproducibility of sound source coordinates across trials and participants, the listener’s head was immobilized with a chin rest throughout the experiment.

#### Virtual sound approach

When studying the impact of head movements on sound localization, one current approach is to exploit auditory virtual reality. Using head-related transfer function (HRTF), it is possible to present sounds through headphones that appear to originate from different positions in 3D space. Virtual sounds prove useful for generating static and moving auditory sources (Dunai et al. 2011) from multiple positions around the listener. In addition, they have been exploited for studying the contribution of visual information to spatial hearing (Majdak et al. 2011). Nonetheless, it remains a challenge to track and update the 3D virtual position of sounds in real time as a function of head movements (for review, see (Lida 2019). Furthermore, the transfer to more clinical settings remains limited because reproducing reliable virtual sounds with HRTF can be particularly difficult and time consuming when participants use hearing aids or cochlear implants (Majdak et al. 2011).

#### Real sound delivered in a virtual reality environment, our approach

Here, we built from the pioneering approach of Brungart and colleagues (1999), overcoming each of the previous limitations. Our approach makes it possible to position the sound source at any controlled 3D position around the subject (without given access to localization cues). The loudspeaker’s xyz coordinates were controlled online by the computer and used to place the sound source in a predetermined position in space. When referenced to the center of the head, computer-controlled placement of the speaker led to an error below 1 cm across all target positions, all participants and all recording sessions. This proves the efficacy of our speaker positioning method, even without physical head restraints.

Our approach allows studies without time-consuming training for experimenters and participants. Notably, all experimenters achieved accurate and fast speaker 3D positioning with only a few minutes training (< 5 min). Most importantly, this approach of pointing to a sound source in its near space required no procedural training to perform the task, and less than 7% of trials were rejected for non-compliance with instructions. Indeed, in the study by Bahu and colleagues (2016), despite training the participants to familiarize with the pointing method to the sound sources, participants had difficulty performing the motor task, especially for the rear sound sources. Our ‘simple’ approach is particularly relevant for the eventual aim of applying this same methodology to developmental and clinical populations (for example, this pointing method to sound source has been used without any difficulty with children, (Coudert et al. 2022).

The use of the HMD was motivated by the fact that we wanted to control visual cues from the environment, control the position of the eyes and the head (it is also used for head-center reference frame). It has proven to be a very good way to identify the active listening strategy of participants. The direction of the head movement reveals the portion of space captured when sound is perceived: our participants faced sounds in front of them, or moved their head in back space for rear sounds. This sound space perception does not need further explicit response from the subject (e.g., hand pointing, verbal response). By recording the head direction as a tool for spatial hearing abilities, subject sound localization performance could be easily explored, and it is crucial when dealing with sound spatial abilities in case of hearing impairment, or dealing with sound targets in the far space. The HMD is an object placed on the face, which constitutes a physical obstacle to sound diffusion close to the ears, modifies the HRTF of the head and impacts sound localization for front sound sources. But in active listening condition, the HMD’s effect is attenuated and it no longer alters the auditory spatial processing. Maybe in the future, HMD will be smaller and therefore have less impact on the HRTF.

### Limitations and perspectives

*Age range of participants.* In this study, we deliberately spread out the age range of the participants. Our objective was to assess the feasibility of our approach in both young and older adults, as this may prove useful when assessing spatial localization ability in in hearing impaired populations. We reached our goal as all the participants included in this protocol followed the instructions and attended the 40-min experiment. However, performance variability was likely introduced into the group of participants. The ability to process auditory spatial information changes over the lifetime and auditory localization accuracy deteriorates in older adults (see Freigang et al. 2015).

When participants responded to the emitted sound by holding their hand at the perceived sound position, no visual feedback of the hand position was given. We limited visual cues from the environment as we wanted to avoid visuo-motor training effect that could be used by the subject to modify his head strategy and/or auditory perception of sound localization. As discussed by Ahrens and colleagues (2019), providing visual information might help to learn possible source locations, which can improve localization accuracy. Moreover, visual cues could influence sound localization abilities: minimal visual spatial frame benefits sound localization task (Valzolgher et al. 2020a, b), a reaching to sound localization task coupled with visual feedback modified head-movement behavior and improves sound localization performance (Valzolgher, et al. 2020a, b). Future studies could manipulate the visual scene and/or the vision of the hand to answer the multifactorial nature of spatial audition.

Finally, we wanted to examine the 3D sound localization performance in the reaching space. This space is actually quite relevant for humans: the near-field portion of space is particularly relevant for social interactions, where fast motor responses are needed in case of an approaching auditory object (e.g., a mosquito), when reaching toward a sound source (e.g., our phone ringing) or when orienting toward a nearby talker. Noticeably, a recent study (Valzolgher et al. 2020a, b) has shown that the ability to interact with a sound in the reaching space improves localization performance and promotes head movements, and this interaction also benefits spatial hearing rehabilitation (Valzolgher et al. 2022). As the participants localize sound sources manually, far-field stimulation is not feasible with our actual setup. However, by adapting the response method (i.e., use of a virtual pointer instead of hand-reaching, or by measuring head-direction as in Valzolgher et al. 2020a, b), this limitation could be addressed.

## Conclusion

Researchers agree on the general notion that spatial hearing is an active and multisensory task. However, this awareness led to little adjustments to the methodological approach typically used when studying this fundamental perceptual ability. For instance, the study of head movements in sound localization remained largely overlooked. One reason for this discrepancy may reside in the fact that considering head movement has been problematic for most approaches to sound localization. In studies relying on sounds delivered in a real environment, head movements have mostly been prevented, to ensure reproducibility of sound source position across trials and participants, or remained uncontrolled. When virtual sounds were generated through HRTFs, implementation of head movement responses in real time is still a computationally challenging task.

The approach we proposed and tested in the present study (SPHERE, European patent n° EP 3,463,084 A1, (Salemme et al. 2021) is a valid tool to accurately sample spatial abilities in auditory perception all around the listener, with minimal constraints on the participant or experimenter. Most interestingly, SPHERE proved sensitive for detecting and quantifying the contribution of free head motion during sound emission, with improvements to sound localization accuracy and precision. The SPHERE approach has been used recently with adult and pediatric populations, on both normal-hearing participants and cochlear implant patients (Coudert et al. 2022; Valzolgher, et al. 2020a, b, 2020a; Valzolgher et al. 2022). It offers a highly versatile opportunity to assess normal and pathological sound localization performance in a more ecologically valid approach (for discussion see Russell 2022). Finally, our approach paves the way for future research, clinical and industrial applications that will leverage the full potential offered by having embedded a VR HMD in the SPHERE system.
